# Marsupial vena cava mimicking lymph node enlargement on tomography

**DOI:** 10.1590/S1679-45082018AI4394

**Published:** 2018-09-10

**Authors:** Antonio José Souza Reis, Marcelo Assis Rocha, George Ramos Lemos, Fernando Ide Yamauchi, Adriano Tachibana, Ronaldo Hueb Baroni

**Affiliations:** 1 Hospital Israelita Albert Einstein, São Paulo, SP, Brazil

**Figure 1:**
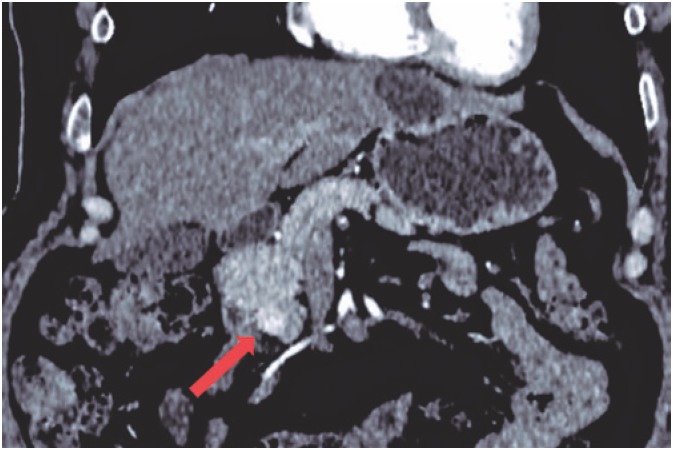
Pancreatic nodule. Coronal contrast-enhanced tomography in the arterial phase demonstrating a hypervascular nodule with neuroendocrine lesion features on the pancreas head (arrow)

**Figure 2:**
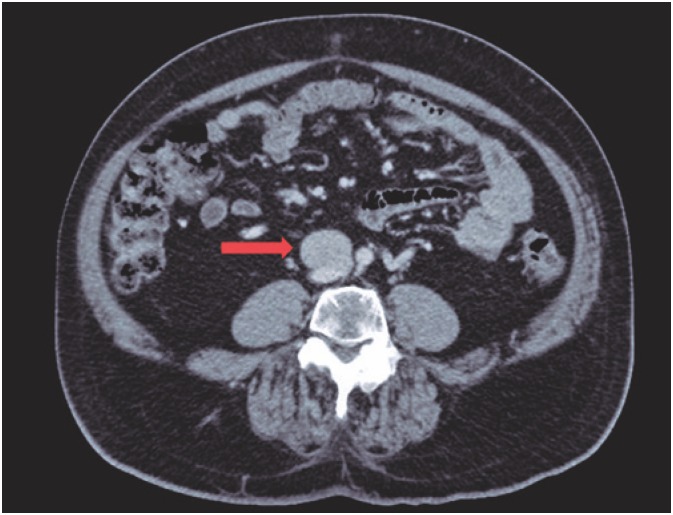
Pre-aortic oval formation. Axial contrast-enhanced tomography in the portal phase demonstrating an oval formation anteriorly to the aortoiliac bifurcation (arrow)

A 56-year-old male patient, hypertensive, diabetic and asymptomatic, underwent abdominal computed tomography (CT) with intravenous contrast for follow-up of hepatic steatosis. Computed tomography demonstrated a solid hypervascular nodule in the pancreatic head (characteristics of neuroendocrine lesion - figure 1) and oval formation anteriorly to the aortoiliac bifurcation (Figure 2).

**Figure 3:**
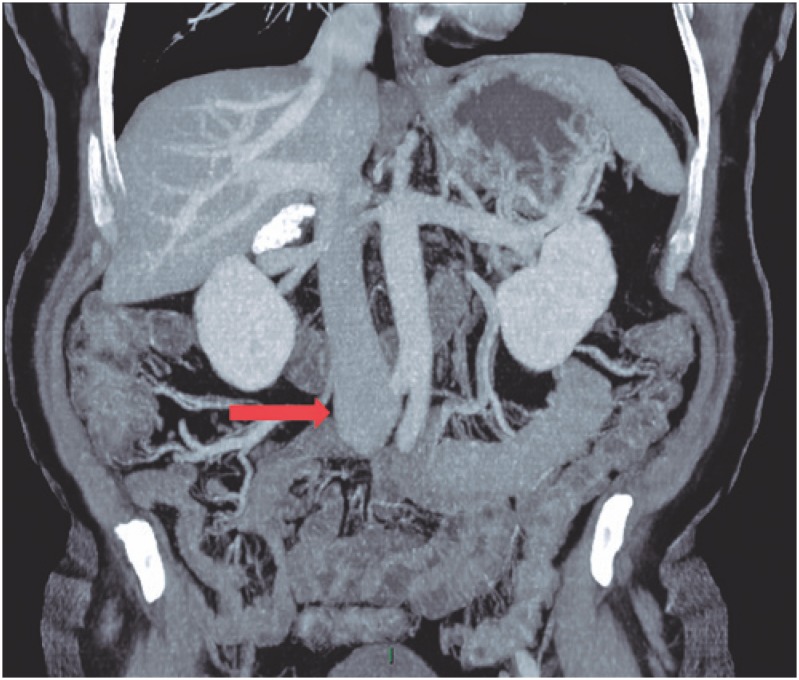
Anomalous inferior vena cava. Coronal contrast-enhanced tomography with MIP in the portal phase showing that the structure anteriorly to the aortoiliac bifurcation is an anomalous course of the inferior vena cava (arrow)

**Figure 4:**
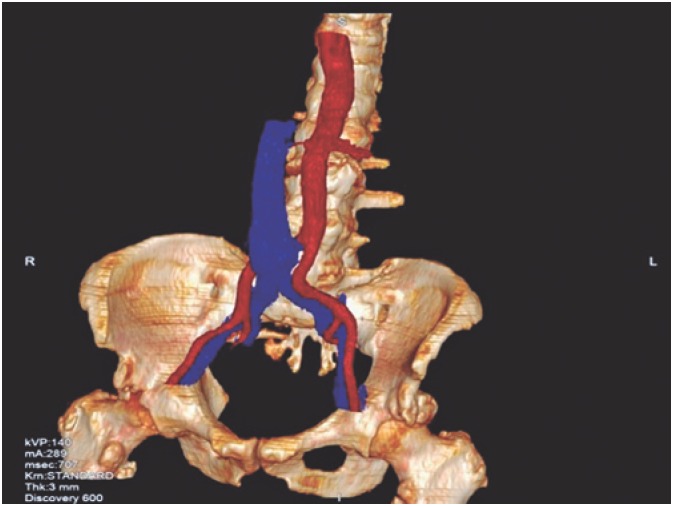
Marsupial vena cava. Tridimensional reconstruction of tomography showing the inferior vena cava (in blue) anteriorly to the aortoiliac bifurcation (in red)

In this situation, axial CT can mimick lymph node enlargement, especially in clinical oncology context. However, evaluation of the different phases of the exam and the reformatted coronal and sagittal images help making the correct diagnosis of a vascular anatomical variation: an anomalous course of the inferior vena cava (IVC) anteriorly to the aortoiliac bifurcation (Figures 3 and 4).

The IVC embryogenesis consists of regressions, anastomoses and substitutions of fetal precursors, and finally the IVC is converted into a unilateral structure, positioned on the right side of the abdomen, comprising four segments: hepatic, suprarenal, renal and infrarenal.^(^ ^1^ ^,^ ^2^ ^)^ Aberrant events in this period determine development anomalies in this system, resulting in 14 different anatomical variations.^(^ ^2^ ^)^ The most common anomalies are duplicated IVC and its positioning on the left side of the abdomen.^(^ ^1^ ^,^ ^3^ ^)^ Others are eventually identified, such as preaortic iliac confluence, known as marsupial vena cava.^(^ ^4^ ^)^

Marsupial vena cava is a congenital anomaly in which the IVC or the left common iliac vein are located anteriorly to the aortic bifurcation or right common iliac artery.^(^ ^1^ ^,^ ^5^ ^)^ This presentation probably represents persistence of the ventral segment of the aortic venous ring, associated to regression of the dorsal segment of this ring. This situation is opposite to the expected normal development.^(^ ^5^ ^)^

Although some complications, such as deep venous thrombosis, may occur, most anomalies of the IVC are asymptomatic.^(^ ^2^ ^)^ Nonetheless, they can lead to misinterpretation during imaging examinations when mixed up with retroperitoneal lesions.^(^ ^2^ ^)^ It is useful to identify them to plan vascular and surgical interventions.^(^ ^2^ ^)^
